# Chronic exposure to tumor necrosis factor alpha induces retinal pigment epithelium cell dedifferentiation

**DOI:** 10.1186/s12974-018-1106-8

**Published:** 2018-03-16

**Authors:** Sara Touhami, Fanny Beguier, Sébastien Augustin, Hugo Charles-Messance, Lucile Vignaud, Emeline F. Nandrot, Sacha Reichman, Valérie Forster, Thibaud Mathis, José-Alain Sahel, Bahram Bodaghi, Xavier Guillonneau, Florian Sennlaub

**Affiliations:** 1 Sorbonne Université, INSERM, CNRS, Institut de la Vision, 17 rue Moreau, F-75012 Paris, France; 2 Department of Ophthalmology, Reference Center in Rare diseases, DHU Sight Restore, Hôpital Pitié Salpêtrière, University Paris VI, 47-83 Boulevard de l’Hôpital, 75013 Paris, France; 3 CHNO des Quinze-Vingts, DHU Sight Restore, INSERM-DGOS CIC 1423, 28 rue de Charenton, F-75012 Paris, France

**Keywords:** Retinal pigment epithelium, Tumor necrosis factor alpha, Transforming growth factor beta, Age-related macular degeneration, Neuroinflammation, Neurodegenerative disease

## Abstract

**Background:**

The retinal pigment epithelium (RPE) is a monolayer of pigmented cells with important barrier and immuno-suppressive functions in the eye. We have previously shown that acute stimulation of RPE cells by tumor necrosis factor alpha (TNFα) downregulates the expression of OTX2 (Orthodenticle homeobox 2) and dependent RPE genes. We here investigated the long-term effects of TNFα on RPE cell morphology and key functions in vitro.

**Methods:**

Primary porcine RPE cells were exposed to TNFα (at 0.8, 4, or 20 ng/ml per day) for 10 days. RPE cell morphology, phagocytosis, barrier- and immunosuppressive-functions were assessed.

**Results:**

Chronic (10 days) exposure of primary RPE cells to TNFα increases RPE cell size and polynucleation, decreases visual cycle gene expression, impedes RPE tight-junction organization and transepithelial resistance, and decreases the immunosuppressive capacities of the RPE. TNFα-induced morphological- and transepithelial-resistance changes were prevented by concomitant Transforming Growth Factor β inhibition.

**Conclusions:**

Our results indicate that chronic TNFα-exposure is sufficient to alter RPE morphology and impede cardinal features that define the differentiated state of RPE cells with striking similarities to the alterations that are observed with age in neurodegenerative diseases such as age-related macular degeneration.

## Background

The retinal pigment epithelium (RPE) is a monolayer of polarized pigmented cells, located between the photoreceptor outer segments (POS) and the choroid. These highly differentiated cells play a crucial role in the visual cycle, the recycling of POS, the formation of the outer blood-retinal barrier [1], and in providing immunosuppressive signals that eliminate infiltrating immune cells from the subretinal space [2].

With age, RPE cells decline in number and the percentage of multinucleated RPE cells increases [3, 4]. Enlarged, irregularly shaped, multinucleated RPE cells are also found in increased numbers in the vicinity of drusen that define early intermediate age-related macular degeneration (AMD) [5]. AMD is also associated with dysfunctional RPE: the visual cycle is slowed, evidenced by an increase of recovery time after bleach [6, 7]; the outer blood-retinal barrier is breached, illustrated by subretinal neovascularization, edema, and macrophages (Mϕ) that accumulate subretinally around the RPE [2]. At later stages the RPE degenerates, forming atrophic zones that define geographic atrophy (GA), the untreatable late form of AMD. Although it seems evident that RPE dysfunction and death is a pivotal part of AMD pathogenesis, it is not yet clear what leads to and aggravates the decay.

We and others previously showed that Mϕs accumulate on the apical side of the RPE adjacent to the atrophic zones that define GA, around choroidal neovascularizations (CNV) and around large drusen in intermediate AMD [2, 8–12]. Using a co-culture model, we recently demonstrated that Mϕ-derived tumor necrosis factor alpha (TNFα) acutely downregulates the transcription factor orthodenticle homeobox 2 (OTX2) that is constitutively expressed in RPE cells, where it controls the expression of a number of essential genes. TNFα is a potent inflammatory cytokine, mainly produced by activated Mϕs and T-cells, with a broad range of biological activities [13]. A possible role of TNFα in age- and AMD-related RPE changes is also suggested by the observation that plasmatic TNFα concentrations increase with age [14, 15] and are further increased in complement factor H (CFH) AMD-risk variant carriers [16]. Furthermore, TNFα expression in monocyte-derived Mϕs increases with the age of the donor [17, 18] and AMD patients whose monocyte-derived Mϕs express the greatest amount of TNFα have a higher prevalence of CNV [19].

We here show that chronic (10 days) exposure of primary RPE cells to TNFα increases RPE cell size and polynucleation, decreases visual cycle gene expression, impedes RPE tight-junction organization and transepithelial resistance, and decreases the immunosuppressive capacities of the RPE. Some, but not all, of these effects were due to TNFα-induced transforming growth factor ß expression.

Taken together our results indicate that chronic TNFα exposure is sufficient to alter RPE morphology and impede cardinal features that define the differentiated state of RPE cells with striking similarities to RPE alterations with age and dysfunctions observed in AMD.

## Methods

### RPE cell-cultures

Isolation of primary porcine RPE cells was conducted according to a previously described protocol [20]. This procedure adheres to the European initiative for restricting animal experimentation because not a single animal was killed for our experimentation specifically. Briefly, porcine eyes were purchased from a local slaughterhouse (Guy Harang, Houdan, France) in agreement with the local regulatory department and veterinarians and transported in CO2-independent medium (Thermo Fisher Scientific) 2 or 3 h after enucleation. Eyes were dissected and cleaned off surrounding cunjunctiva, tenon’s capsule, and muscles then immersed (2 × 5 minutes) in an antiseptic solution (Pursept-A Xpress, Merz Hygiene GmbH). The anterior segment of the bulb including the lens, vitreous gel, and the retina were then removed to isolate an eye-cup made of sclera, choroidal, and RPE cells. Each eye cup was washed twice with PBS (Thermo Fisher Scientific) and incubated with 0.25% trypsin–EDTA (Thermo Fisher Scientific) for 75 min at 37 °C. Trypsin-detached RPE cells were pipetted off the choroid and resuspended in Dulbecco’s modified Eagle’s medium (DMEM, Thermo Fisher Scientific) supplemented with decomplemented 20% fetal calf serum (FCS, Thermo Fisher Scientific) and 1% penicillin/streptomycin (PS) (final concentrations 100 U/ml and 100 μg/ml) antibiotic mixture. Purified cells were seeded in 60-mm Petri dishes in DMEM-FCS20%-PS1% and incubated in a 5% CO2 regulated atmosphere at 37 °C. The culture DMEM-FCS20%-PS1% medium was changed after 24 h. When the cells reached a 70–80% confluence state (usually at day 4), 0.05% trypsin–EDTA was added for 5 min at 37 °C. The cells were then seeded on either 96-well culture plates (Corning) at the concentration of 75,000 cells/well or in 6.5 mm trans-well polyester filters (0.4 μm pore size, Corning, Kennebunk, ME, USA) at a concentration of 100,000 cells/well in DMEM-FCS20%-PS1%. Visual confluence was obtained after 2 weeks in culture at 37 °C, 5% CO2, and cells were used immediately for TNFα treatment.

### Human blood monocyte isolation and supernatant preparation

Human blood from healthy donors was collected for monocyte isolation after written informed consent in the Centre National d’Ophtalmologie des Quinze-Vingts (Paris). The protocol was approved by the *Direction Générale pour la Recherche et l’Innovation* of the *Ministère de l’Enseignement et de la Recherche* (Dossier n°14.007) and by the *Commission Nationale de l’Informatique et des Libertés* (N/Ref.: IFP/MKE/AR144088). Briefly, peripheral blood mononuclear cells were obtained by Ficoll gradient centrifugation of fresh human blood, washed three times with PBS, and negatively sorted using the EasyStep Human Monocyte Enrichment Cocktail without CD16 Depletion kit (StemCell Technologies).

Lipopolysaccharide (LPS, O127:B8)-stimulated monocyte (Mo) supernatant was produced by a 2-h stimulation of freshly prepared Mo, followed by a 24 culture of 100,000 Mo in 0.1 ml of fresh DMEM containing 1%penicillin/streptomycin (without LPS). The SN were then stored at − 80 °C until use in RPE cultures.

## Culture protocols and cell treatments

### RPE monoculture

RPE cells were kept in culture until confluence then serum starved the day before starting supernatant (SN), TNFα and/or anti TGFβ treatments, in order to minimize the effects of serum on culture outcomes. In supernatant experiments, Mo SN was added daily for a maximum duration of 10 days. In TNFα experiments, recombinant TNFα (from R&D) was added daily in 1%FCS-1%PS DMEM at various concentrations: 0.8, 4, or 20 ng/ml for 10 days. In inhibition experiments, anti-TGFβ (Sigma, ref. SB505124, 500 nM) was added daily and simultaneously with TNFα (in 1%FCS-1%PS DMEM), for 10 days. At the end of culture, cells were washed twice with PBS and fixed in 4% paraformaldehyde (PFA) for 10 min. Alternatively, RA1 buffer was added to the cells to perform real-time quantitative polymerase chain reaction (RT-qPCR).

### RPE-monocyte coculture

To evaluate the possible changes in their immunosuppressive capacity, RPE cells were pretreated during 10 days in 1%FCS-1%PS DMEM +/− different concentrations of recombinant TNFα before adding freshly purified human monocytes. For coculture purposes, RPE cells were serum and TNFα starved the day before adding 100,000 monocytes to RPE cells in each well. Cells were incubated at 37 °C for a total of 24 h.

### Immunofluorescence microscopy

At the end of culture, cells were fixed in 4% PFA for 10 min then washed twice in PBS and incubated for 2 min in a permeabilizing solution (0.1% triton- 0.1% sodium citrate in PBS). Cells were then blocked for 1 h in PBS-0.1% triton-5% horse serum (Thermo Fisher Scientific) and incubated overnight at 4 °C with the following primary antibodies: polyclonal goat anti-human OTX2, 1/500, R&D; monoclonal rat anti-mouse Zonula Occludens(ZO)-1, 1/200, Millipore and rabbit anti-human hematopoietic transcription factor PU-1, 1/200 diluted in PBS triton 0.1% and 1% horse serum. Alexa fluor 594 phalloidin (Invitrogen) was used for F-actin staining. Secondary antibodies produced in donkey were used at room temperature for 1 h (AlexaFluor 488 nm and 647 nm, 1/500, Thermo Fisher Scientific), and nuclei were counterstained with Hoechst (1/1000, Sigma-Aldrich). Cells were washed twice in PBS and observed under fluorescence microscope (Arrayscan VTI HCS Reader, Thermo Fisher Scientific). Twenty-five fields per well were analyzed and recorded using the Arrayscan software (HCS iDev Cell Analysis Software, Thermo Fisher Scientific).

### Measurement of transepithelial resistance

Transepithelial resistance of RPE cells grown on trans-well inserts was measured daily using a WPI EVOM (World Precision Instruments, Sarasota, FL, USA) device. All measurements were performed in the cell culture hood within 5 min of removal from the incubator. For TER measurement purposes, the measuring probe was immersed beforehand in 100% ethanol for 15 min then incubated in culture medium for two additional hours. Net TER was obtained by subtracting the value of a trans-well filter without cells from that of cell-containing filters. The TER per unit area (Ω cm^2^) was obtained by multiplying the raw TER value by the surface of trans-well inserts (0.33 cm^2^). Before the start of TNFα or anti-TGFβ treatments, the polarization of RPE cells was determined and confirmed by their high TER (> 250 Ω cm^2^). All measurements were expressed as percentage of control.

### Phagocytosis assay

RPE phagocytosis of porcine photoreceptor outer segments (POS) was evaluated according to a previously described protocol [21]. RPE cells were pre-treated in 1%FCS-1%PS DMEM in presence of different concentrations of TNFα for 10 days then serum starved 24 h before evaluating their POS phagocytic capacity (exposure to POS: 3 h).

### Western blot analysis

Rhodopsin degradation was analyzed qualitatively in a Western blot (WB) assay. Briefly, RPE cells were previously cultured in 1%FCS-1%PS DMEM, with or without different concentrations of TNFα for 10 days, then serum starved before adding the POS (according to a previously described protocol [21]) for 6 and 24 h respectively. After these incubation periods, supernatants were removed and RIPA buffer was added to RPE cells before performing the WB as previously described [22] using primary anti-Rhodopsin (Merck ref MAB5316) and anti-β-actin antibodies (Sigma).

### Proliferation assay

DNA replication in RPE cells was assessed in vitro using the Click-it EDU (5-ethynyl-2′-deoxyuridine) Alexa Fluor 594 Imaging kit according to the manufacturer’s instructions after fixating the cells in 4% PFA for 10 min and preparing them for immunofluorescence microscopy using primary and secondary antibodies as described earlier.

### Cell-fusion study

Freshly collected porcine RPE cells were incubated for 20 min at 37 °C in presence of either 5 μM CFSE or 1 μM Far red CFSE (cell trace, cell proliferation kits, Invitrogen) according to the manufacturer’s protocol then kept for 5 min in 20%FCS 1%PS DMEM and centrifuged at 800 rpm for five additional minutes. The cells were then seeded in 16-well glass slide lab-teks (Nunc) at the final concentration of 75,000 cells per well in DMEM-FCS20%-PS1% (including 50% CFSE labeled and 50% Far red CFSE labeled cells). When confluence was reached, cells were serum starved and treated daily with either DMEM-PS1% or TNFα-enriched medium as previously described.

### Reverse transcription and real-time quantitative polymerase chain reaction

Total RNA from cell cultures was extracted using the Nucleospin RNAII extraction kit according to the manufacturer’s protocol (Macherey Nagel). RNA yields were then measured using the NanoDrop™ 8000 spectrophotometer at a 260 nm wavelength. Overall, RNA concentrations ranged between 40 and 80 ng/μL. Reverse transcription into single-strand cDNA was performed using 1 μg of total mRNA (pretreated with DNase), oligo-dT, and superscript II reverse transcriptase (Thermo Fisher Scientific). For real-time PCR, 1/50 of cDNA was incubated with the polymerase and appropriate amounts of nucleotides (PowerSYBR Green PCR mix, Applied Biosystems) and PCR was performed using StepOne Plus real-time PCR system (Applied Biosystems). Results were normalized using house-keeping gene RPS26. PCRs were performed in 45 cycles of 15 s at 95 °C, 45 s at 60 °C. Primers for RT-PCR were purchased from IDT technology. Sequences are as follows: ACTA2 forward 5′-CCAACCGGGAGAAGATGACC- 3′; ACTA 2 reverse 5′- AGAGTCCAGCACAATGCCAG- 3′; CLD19 forward 5’-GCCCTAGCACACCTGTCAAT -3′; CLD19 reverse 5′- ACGTGCAGCAGAGGAACGAG-3′; OCLD forward 5’-ATTTATGACGAGCAGCCCCC-3′; OCLD reverse 5’-GCATAGTCCGAAAGGGGAGG-3′; RPE65 forward 5′- TTCTCTTTTCAGGGCCTCGT-3′; RPE65 reverse 5′- AAAGATGGGTTCGGATGGGT-3′; RPS26 forward 5’-TCGATGCCTATGTGCTTCCC-3′; RPS26 reverse 5’-CAGCACCCGCAGGTCTAAAT-3′; TGFβ1 forward: TTACAACAGTACCCGCGACC; TGFβ1 reverse: CCGCTTTCCAGCATTAGCAC; TTR forward 5′- TGGAAGGCACTTGGCATTTC-3′; TTR reverse 5′- GGTGGAGTAAGAGTAGGGGC -3′.

### Statistical analyses

Graph Pad Prism 7 (GraphPad Software) was used for data analysis and graphic representation. All values are reported as mean ± SEM. Statistical analyses were performed by one-way ANOVA analysis of variance, Student *t* test or Mann–Whitney test for comparison of mean values when applicable, followed by Bonferroni post-test. The *p* values are indicated in the figure legends.

## Results

### Chronic exposure to activated monocyte supernatants or TNFα increases the percentage of enlarged, multinucleated RPE cells

**Fig. 1:**
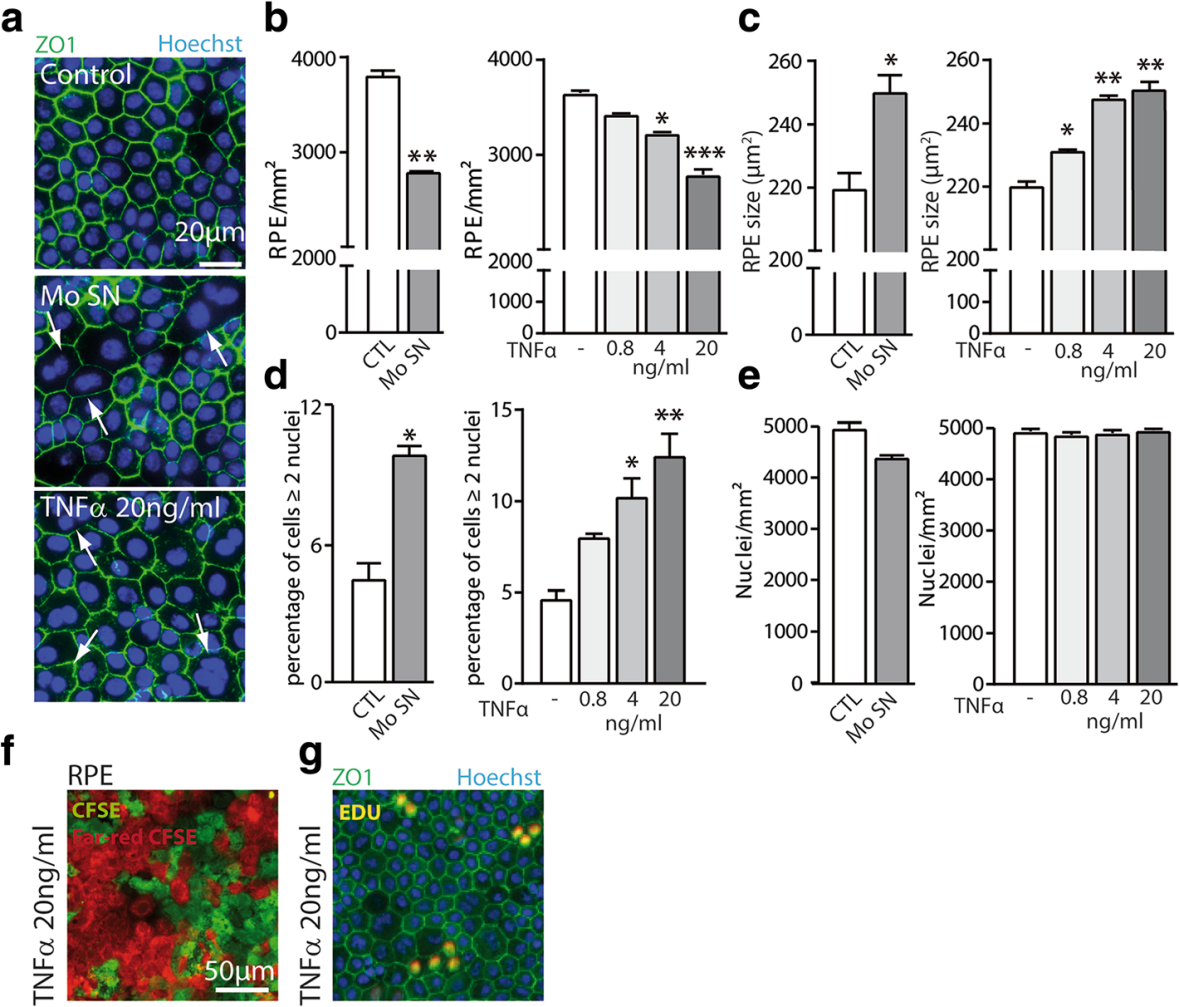
Chronic exposure to activated monocyte supernatants or TNFα increases the percentage of enlarged, multinucleated RPE cells. Porcine retinal pigment epithelium (RPE) cells were cultured with or without lipopolysaccharide activated monocyte supernatants (Mo SN) or TNFα added daily at different concentrations for a total of 10 days. **a** Representative pictures of Zonula Occludens (ZO)1 (green) and Hoechst (blue) immunohistochemistry of control, Mo SN, and TNFα-exposed RPE. Automated quantifications by Arrayscan of **b** the number of RPE cells, **c** RPE cell size, **d** the number of multinucleated RPE cells (≥ 2 nuclei), and **e** total number of nuclei per mm^**2**^ in the indicated different conditions. Representative images of 10-day TNFα-exposed RPE cell-cultures of **f** a co-culture of 50% RPE cells labeled with Carboxyfluorescein succinimidyl ester (CFSE) (green) and 50% RPE cells labeled with Far-red CFSE (red), and **g** ZO-1- (green) and Hoechst nuclear- (blue) stain of cultures maintained in the presence of EDU (5-ethynyl-2′-deoxyuridine, red). (*n* = 5/group, one way ANOVA or Mann-Whitney, comparison versus control: *P < 0.05, **P < 0.005*** *P* < 0.0005)

In vitro, confluent primary porcine RPE cells form a mosaic of hexagonal and regularly shaped cells with tight junctions that can be visualized by a Zonula Occludens 1 (ZO1) immunohistochemistry (Fig. 1a). Ten daily repeated exposures to lipopolysaccharide (LPS)-stimulated Mo supernatant (Mo SN) or daily exogenously added TNFα did not induce RPE cell monolayer defects but led to enlarged and irregularly shaped RPE cells (Fig. 1a). Automated quantification of the number of ZO1-demarcated RPE cells using an Arrayscan revealed that Mo SN exposure decreased the cell number (Fig. 1b) in parallel to an increase of average RPE cell size (Fig. 1c). TNFα stimulation was sufficient to induce a similar effect in a dose-dependent manner (Fig. 1b, c). The increase in RPE cell size was associated with a significant increase of the percentage of polynucleated cells in both conditions (Fig. 1a arrows and Fig. 1d) that led to a complete compensation of the overall density of nuclei per surface (Fig. 1e). Ten-day culture of 50% CFSE-stained and 50% CFSE far red-stained RPE cells showed no red/green double-labeled cells in TNFα-exposed cell-cultures, excluding cell fusion as a mechanism of polynucleation in this condition (Fig. 1f). Addition and revelation of the traceable nucleotide 5-ethynyl-2′-deoxyuridine (EDU) to the TNFα-exposed cell-cultures showed that nuclei of multinucleated RPE cells were EDU positive (Fig. 1g), demonstrating that polynucleation was due to nuclei replication and failed cytokinesis as previously shown in aged mice in vivo [3].

In conclusion, 10-day exposure to Mo SN or TNFα alone provided a sufficient stimulus to induce an increase in the percentage of enlarged, multinucleated RPE cells similar to what is observed in vivo with age [3, 4].

### Chronic TNFα decreases visual cycle gene expression in the RPE

**Fig. 2:**
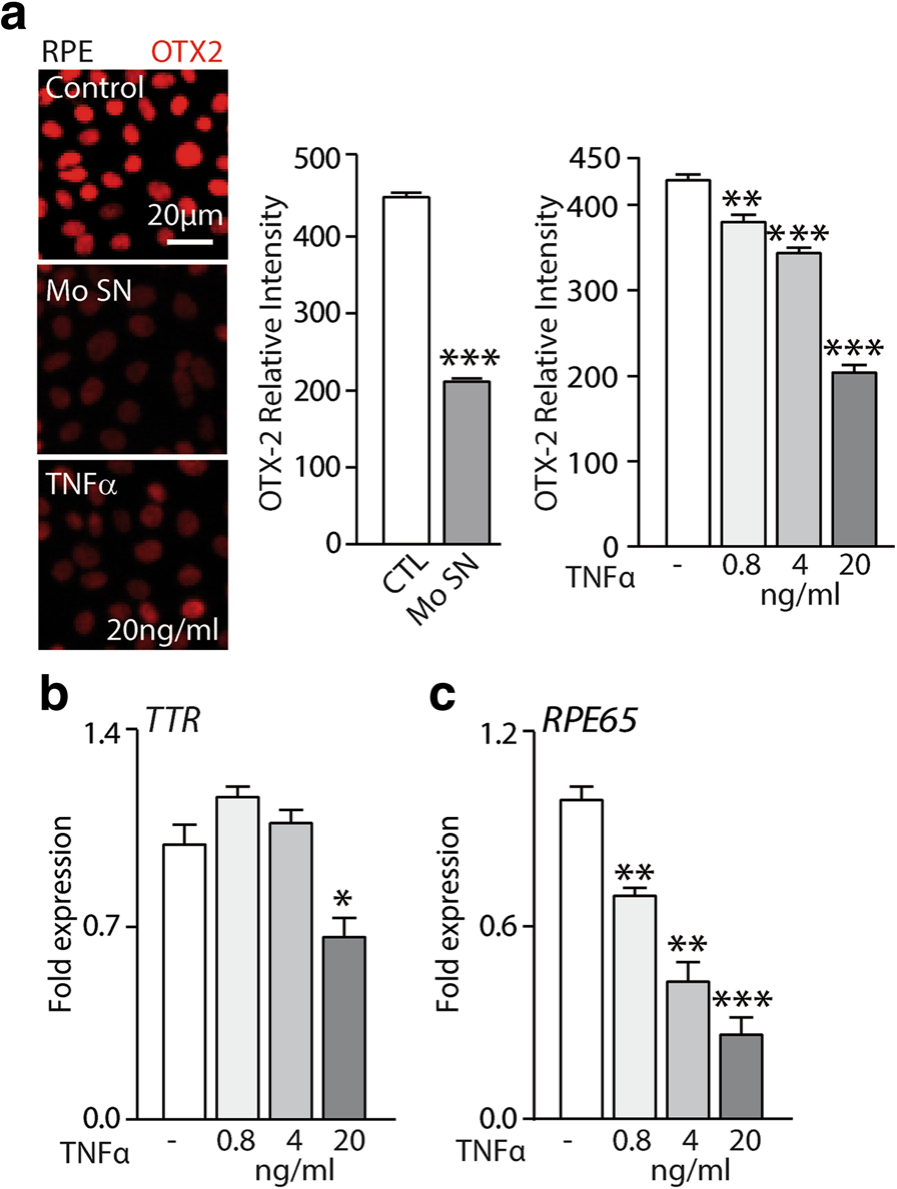
Chronic exposure to activated monocyte supernatants or TNFα decreases visual cycle gene expression in the RPE. **a** Representative pictures of RPE staining with OTX2 (Orthodenticle homeobox 2, red) antibody and Arrayscan fluorescence intensity quantification of OTX-2 staining after 10 days of culture with or without lipopolysaccharide-activated monocyte supernatants (Mo SN) or TNFα added daily at different concentrations. **b** TTR (transthyretin) and **c** RPE65 (retinal pigment epithelium specific *65* kDa protein)-mRNA expression normalized with S26 expression quantified by RT-qPCR of 10-day RPE culture exposed to the indicated TNFα concentrations (*n* = 5/group one-way ANOVA or Mann-Whitney, comparison versus control * *P* < 0.05,**P < 0.005, ***P < 0.0005)

Transthyretin (TTR), a retinol carrier, and RPE65 that is responsible for the conversion of all-trans-retinyl esters to 11-cis-retinol are strongly expressed by the RPE and essential parts of the visual cycle. We have previously shown that TNFα acutely reduces the expression of TTR and orthodenticle homeobox 2 (OTX2) that regulates its expression [22]. Here, repeated stimulation of primary RPE by TNFα diminished the intensity of OTX2-staining measured with an Arrayscan similar to 10 repeated Mo SN exposures (Fig. 2a). Chronic TNFα exposure also significantly reduced TTR transcription, measured by RT-qPCR, at higher concentrations (Fig. 2b). Additionally, we noticed a dose-dependent decrease of RPE65 transcription (Fig. 2c).

In summary, we demonstrated that chronic exposure to Mo SN and TNFα downregulated the expression of major RPE visual cycle genes.

### Chronic exposure to TNFα disrupts RPE barrier properties

**Fig. 3:**
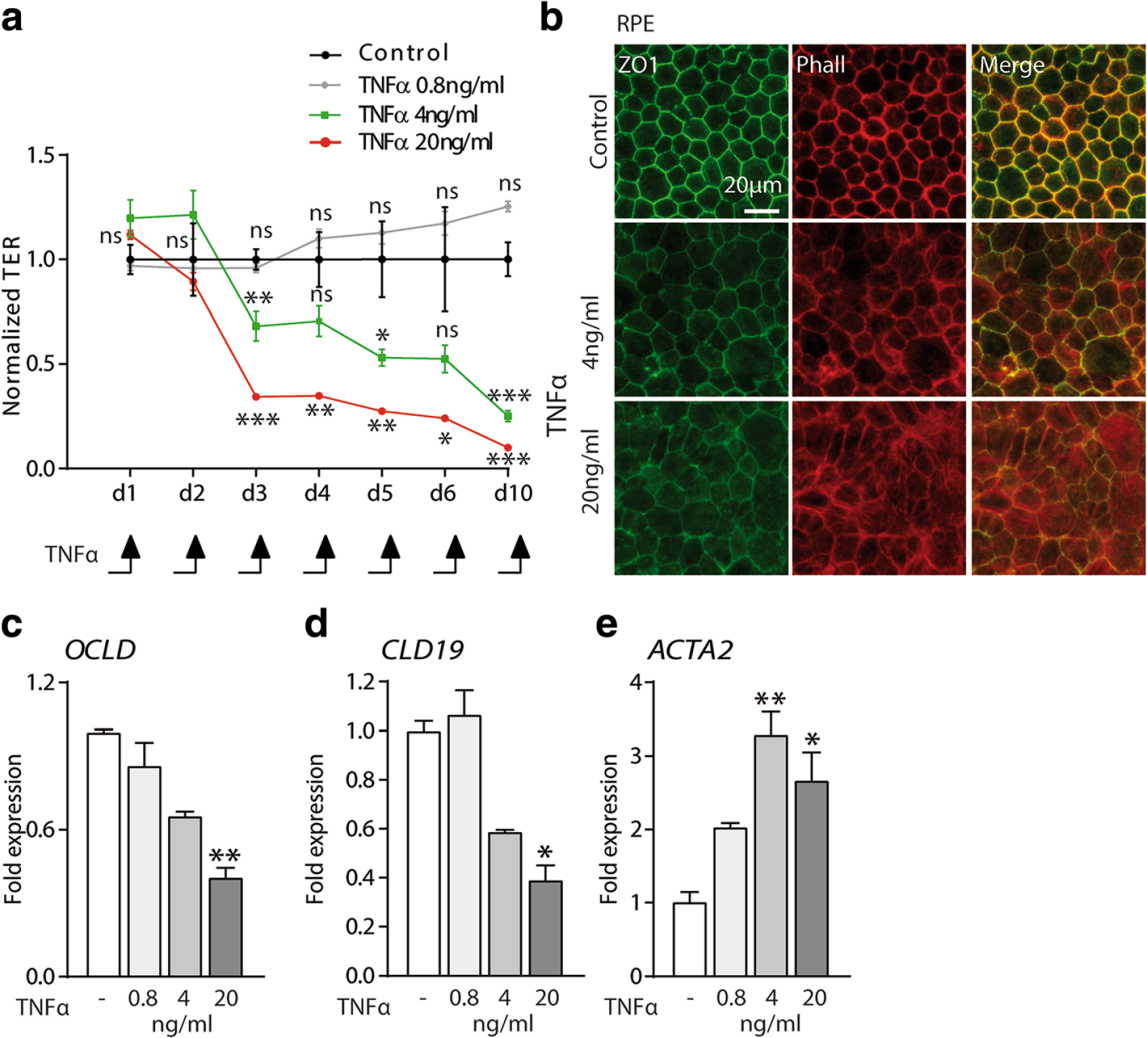
Chronic exposure to TNFα disrupts RPE barrier properties. **a** Measurements of RPE transepithelial electric resistance (TER) measured daily in a 10-day trans-well culture with or without different concentrations of TNFα (*n* = 3/group, one-way ANOVA, versus control for each time point *P < 0.05, **P < 0.005, ***P < 0.0005). **b** Representative pictures of RPE staining with Zonula occludens(ZO) 1 (green) and F actin (Phalloidin, red). **c**–**e** Relative expression of OCLD (Occludin), CLD19 (Claudin-19), and ACTA2 (Smooth muscle alpha (a)-2 actin) mRNA expression normalized with S26 quantified by RT**-**qPCR of 10-day RPE culture with or without the indicated TNFα concentrations (*n* = 5/group one-way ANOVA, versus control *P < 0.05, **P < 0.005, ***P < 0.0005)

Physiologically, individual RPE cells are joint by tight junctions (TJ) to form an ion-impermeable epithelium that constitutes the outer blood-retinal barrier from the fenestrated vessels of the choroid. In vitro, this RPE function is reflected by a high transepithelial electrical resistance (TER) [23]. Repeated TNFα exposure of confluent primary RPE cells grown in trans-wells significantly diminished the TER in a dose- and time-dependent manner starting at day 3 (Fig. 3a). Immunohistochemistry of 10-day TNFα-exposed RPE cells using ZO1 antibody and phalloidin that binds to F actin filaments showed a loss of the preferential location to cell borders of both proteins in the 4 and 20 ng/ml groups (Fig. 3b). Both groups also displayed a significantly inhibited transcription of TJ components occludin and claudin19 by RT-qPCR at day 10 (Fig. 3c, d). On the contrary, repeated exposure to even 0.8 ng/ml of TNFα increased the transcription of smooth muscle alpha (a)-2 actin (ACTA2), a major constituent of the contractile fibers that are classically expressed in myofibroblasts and smooth muscle cells (Fig. 3e).

Taken together, our data shows that repeated exposure to TNFα induces profound changes in cytoskeleton and TJ component expression and leads to the loss of the physiological TER that characterizes primary RPE cells.

### Chronic exposure to TNFα does not alter the phagocytosis and degradation of rod outer segments

**Fig. 4:**
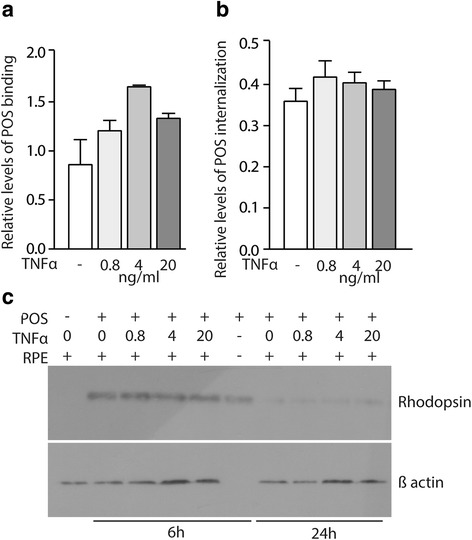
Chronic exposure to TNFα does not alter the phagocytic and rhodopsin degradation capabilities of RPE cells. **a** Quantification of relative levels of photoreceptor outer segment (POS) binding and **b** POS internalization by RPE cells in the indicated culture conditions (*n* = 5/group one-way ANOVA, versus control, non-significant differences). **c** Western blot analysis of Rhodopsin and β-actin protein after 6 and 24 h of culture of primary porcine RPE cells (pretreated with 10-day TNF-α at 0.8, 4, and 20 ng/mL) and photoreceptor outer segments (POS)

Phagocytosis of shed photoreceptor outer segments (POS) and recycling is another important role of RPE cells. To examine the effects of chronic TNFα exposure on RPE phagocytic function, we incubated FITC-labeled POS with control and 10-day TNFα pre-treated RPE cells as previously described [21]. After 3 h of incubation with POS, we noted a tendency to increased POS binding by RPE cells (Fig. 4a), but no difference in the amount of internalized POS between the groups (Fig. 4b). Furthermore, Western blots of rhodopsin of the same protein amount from control and TNFα pre-treated RPE cells, exposed to POS for 6 and 24 h, clearly demonstrated POS degradation after 24 h in both control and TNFα pre-treated cells (Fig. 4c). If normalized to actin ß levels, which increased dose dependently with TNFα levels, rhodopsin remnants at 24 h in TNFα pre-treated cells would seem to be even lower than in the control group (Fig. 4c).

Together, these experiments suggest that chronic TNFα exposure does not impede the RPE’s capacity to phagocytize and digest POS.

### Chronic exposure to TNFα impairs the immunosuppressive RPE cell function

**Fig. 5:**
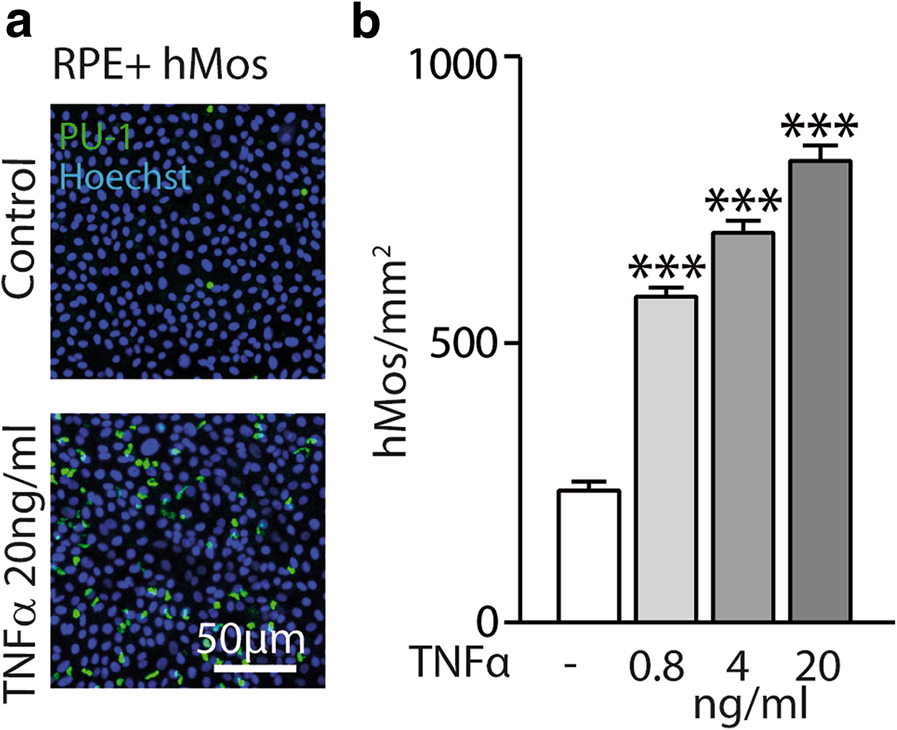
Chronic exposure to TNFα impairs the immunosuppressive function of RPE cells. **a** Representative pictures of human monocytes (hMos, human hematopoietic transcription factor (PU1) staining, green) and RPE nuclei (Hoechst, blue) and **b** automated quantification of the number of human monocytes in a 24-h co-culture with RPE cells with or without previous 10-day pre-treatment with TNFα (at 0.8, 4, and 20 ng/ml) (*n* = 5/group, one way ANOVA versus control ***P < 0.0005)

The subretinal space is an immunosuppressive environment, mediated in part by immunosuppressive signals of the RPE [2]. In vitro, we have recently shown that primary RPE cells rapidly induce the elimination of non-activated monocytes (Mo) in a cell-contact dependent manner [22]. To test whether chronic TNFα exposure altered the RPE’s capacity to eliminate Mo, we incubated equal numbers of peripheral blood Mo with control and 10-day TNFα pre-treated RPE cells for 24 h and stained the co-culture with an anti-PU1-antibody that specifically recognizes Mo, which express this transcription factor, contrary to RPE cells (Fig. 5a). Arrayscan quantification of the number of PU1^+^Mo demonstrates up to a 500% increase in Mo numbers in the co-culture conditions, where the RPE was pre-incubated repeatedly with TNFα (Fig. 5b).

Taken together, our data demonstrates that chronic exposure of RPE to TNFα severely diminishes its immunosuppressive capacities.

### TNFα-induced TGF-ß expression mediates the RPE polynucleation and impairement of barrier properties

TNFα induces the expression and DNA binding of AP-1 (activator protein-1) resulting in increased transcription of the TGF-β1 gene in lung fibroblasts [24]. Furthermore, TGF-β1 has been shown to mediate fibrosis in the lung and to induce RPE dedifferentiation in the context of retinal detachment and proliferative vitreoretinopathy [25].

**Fig. 6:**
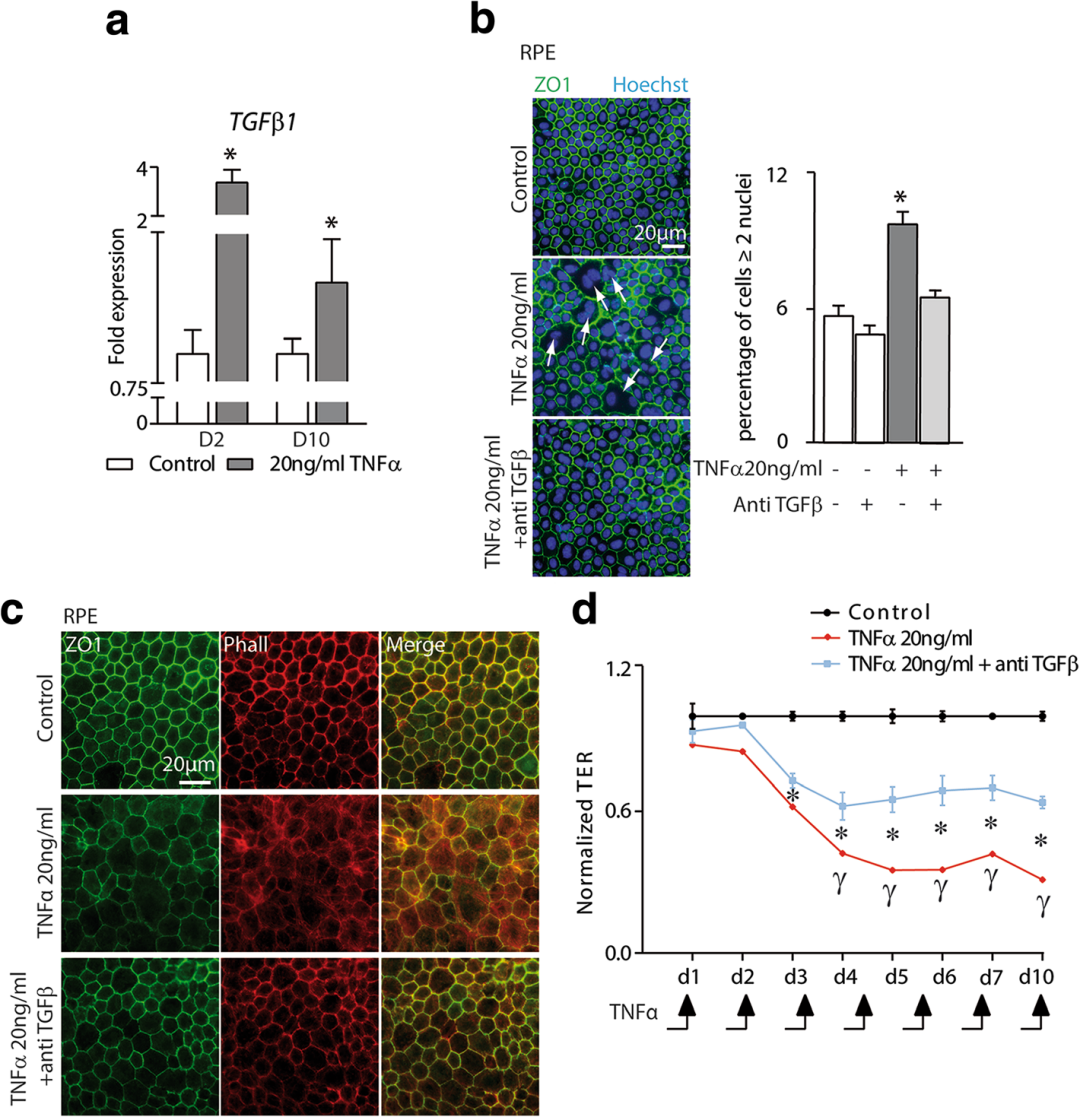
TNFα-induced TGF-ß expression mediates the RPE polynucleation and impairment of barrier properties. **a** TGFβ 1-mRNA expression normalized with S26 expression quantified by RT-qPCR of 2, and 10-day RPE culture exposed to 20 ng/ml of TNFα. **b** Representative pictures of Zonula Occludens (ZO)1 (green) and Hoechst (blue) immunohistochemistry of control, TNFα(20 ng/ml), and TNFα(20 ng/ml) + antiTGFβ (500 nM)-exposed RPE. Quantifications of the number of multinucleated RPE cells (≥ 2 nuclei) in the indicated conditions. (**a**, **b**, *n* = 5/group, one-way ANOVA or Mann-Whitney versus control * *P* < 0.05, **P < 0.005, ***P < 0.0005). **c** Representative pictures of RPE staining with Zonula occludens (ZO) 1 (green) and F actin (Phalloidin, red) after 10 days of culture in the indicated conditions. **d** RPE transepithelial electric resistance (TER) measured daily in a 10-day trans-well culture with or without TNFα (20 ng/ml) and TNFα (20 ng/ml) + antiTGF-β (500 nM) (*n* = 3/group, *P < 0.05 one-way ANOVA of control versus TNFα and TNFα + antiTGF-β for each time point, ϒ *P* < 0.05 Mann-Whitney of TNFα + antiTGF-β versus TNFα for each time point)

RT-qPCR analysis showed that repeated TNFα exposure induced a strong increase of TGFβ1 transcription at 2 days that remained increased after 10 days of culture (Fig. 6a). Interestingly, the co-administration of TGFβ-receptor inhibitor (500 nM) to the RPE culture inhibited the TNFα-induced polynucleation described in Fig. 1 (Fig. 6b). Similarly, TGFβ-receptor inhibitor prevented the TNFα-induced dislocation of the RPE junction protein ZO1 and F-actin (Fig. 6c) and partially restored the TER (Fig. 6d). On the other hand, the inhibition of TGFβ signaling did not prevent the TNFα-induced decrease in OTX2 expression (data not shown).

Taken together, these data strongly suggest that some, but not all, of the TNFα-induced RPE dysfunction is mediated by increased TGF-ß signaling that has previously been shown to perturb certain RPE functions.

## Discussion

We and others previously showed that Mϕs accumulate on the apical side of the RPE adjacent to atrophic zones that define GA, around choroidal neovascularizations and around large drusen in intermediate AMD [2, 8–12, 26]. The RPE is therefore likely chronically exposed to Mϕ-derived cytokines that might alter its function. One of the key cytokines of potential interest is TNFα, which increases with age in plasma and Mϕs [14, 15, 17, 18] and is increased in CFH AMD-risk variant carriers [16]. We recently demonstrated that Mϕ-derived TNF-α acutely downregulates the transcription factor orthodenticle homeobox 2 (OTX2) that is constitutively expressed in RPE cells, where it regulates a number of essential RPE genes [22] and TNF-α exposure has been shown to acutely diminish RPE barrier functions [23]. We here exposed primary RPE cells to 10 daily repeated TNFα stimulations to mimic the exposure of in vivo chronic inflammatory conditions and evaluated its impact on RPE morphology and its main distinctive functions: visual cycle gene expression, tight-junction organization and transepithelial resistance, POS phagocytosis and ROS degradation, and its immunosuppressive capacities. We exposed the RPE to a range of TNFα concentrations from 0.8 to 20 ng/ml, which we administered daily, as TNFα has been reported to have a short half-life of around 15 min [27] and compared some of the findings to the exposure of supernatants from 24 h LPS-activated monocytes (Mo SN).

First, using ZO1/nuclear Hoechst immunohistochemistry, we observed that after 10 days of exposure to Mo SN, the RPE remained confluent. However, the number of RPE cells had decreased significantly, which was compensated by an increased, albeit irregularly shaped, cell-size. In parallel, the percentage of polynucleated cells was significantly increased compared to controls. TNFα exposure was sufficient to induce very similar morphological differences in a dose-dependent manner. TNFα exposure of cultures using a mixture of red- and green-labeled RPE cells and EdU incorporation assays also revealed that the polynucleated cells were not the result of cell fusion but the consequence of nucleic replication in the polynucleated cells. Interestingly, the nucleic replication exactly compensated for the cells lost during the 10-day TNFα and Mo SN exposure, suggesting that the number of nuclei per RPE was finely regulated by their cell size. Interestingly a similar mechanism is also observed in vivo with age [3] that is associated with increased TNFα levels [14, 15, 17, 18] and in the vicinity of large drusen [5]. Although these differences were observed in the 0.8 ng/ml, and 4 ng/ml group, they were most prominent in the 20 ng/ml group and similar to Mo SN exposure.

We next evaluated if chronic TNFα exposure influenced the expression of visual cycle genes. We have previously shown that TNFα acutely downregulates the expression of the transcription factor OTX2 that regulates a number of RPE-specific genes, notably TTR that is involved in the visual cycle [22]. Similarly, chronic TNFα exposure inhibited OTX2 expression, alike to Mo SN exposure, evaluated by immunohistochemistry and fluorescence quantification. It not only diminished the transcription of TTR but also induced a strong and dose dependent reduction in the expression of RPE65 that is not controlled by OTX2. Our observations in primary RPE cells thereby reproduce similar findings in an RPE cell line [28]. Together these results confirm our previous findings and suggest that chronic TNFα exposure reduces the expression of several RPE-specific genes that are implicated in the visual cycle. Dysfunction of the visual cycle leads to an increase of recovery time in the scotopic ERG after bleach, as it takes more time to reproduce 11-cis-retinal. Interestingly, patients with large drusen that are associated with polynucleated RPE cells [5] also present a marked increase in recovery time after bleach [6, 7], which could be due to exposure to TNFα secreted by the associated chronic infiltrate.

Physiologically, individual RPE cells are joint by TJs and the confluent RPE forms an ion-impermeable epithelium that constitutes the outer blood-retinal barrier from the fenestrated vessels of the choroid. We here show that repeated TNF-α exposure of primary RPE induces a dose-dependent loss of the TER, a functional measure of its barrier function, at 4 ng/ml and 20 ng/ml, that worsens with each TNF-α application. Both, 4 ng/ml and 20 ng/ml TNF-α-exposed RPE cells also displayed a loss of the preferential location of the TJ protein ZO1 and F actin filaments to cell borders, diminished transcription of the TJ components occludin and claudin19, and increased expression of smooth muscle alpha (α)-2 actin (ACTA2), in accordance with altered barrier functions. A similar decrease of TER has previously been described after 24 h of a single stimulation with TNF-α at 10 to 50 ng/ml [23], which was not observed after a stimulation with 100 pg/ml [29]. Taken together, our data and the data from the literature suggest that exposure to TNF-α, and in particular repeated exposure, severely impedes RPE barrier functions.

We next evaluated if chronic TNFα-exposure affected the RPE’s capacity to phagocyte and digest POS, an important function for their recycling and renewal. Interestingly, the phagocytosis assay revealed a tendency of increased POS binding, little differences in POS uptake, and no differences in rhodopsin degradation, the major protein of POS. These results suggest that chronic TNFα exposure has little influence on RPE phagocytosis, contrary to the other examined functions.

Last but not least, we examined the immunosuppressive capacity of TNFα pre-exposed RPE by incubating the cells with monocytes after their 10-day cytokine exposure. While monocytes were quickly eliminated by the contact with control RPE for 24 h, as previously described [22], we observed a very significant dose-dependent increase in monocyte survival of the co-culture in TNFα-exposed RPE, suggesting that their capacity to induce monocyte death was severely reduced.

TNFα induces the expression and DNA binding of AP-1 resulting in increased transcription of the TGF-β1 gene in lung fibroblasts [24]. Our results show that it similarly induces TGF-β1 in RPE cells. Interestingly, pharmacological inhibition of TGF-β signaling prevented the TNFα-induced increase in polynucleated RPE cells and reduced the decline of the TER but did not restore OTX2 expression levels. These results suggest that some, but not all, of the TNFα-induced effects are mediated by TGF-β1.

## Conclusions

Taken together our results indicate that chronic TNFα exposure is sufficient to alter RPE morphology and impede three out of four cardinal features that define the differentiated RPE. These alterations bare striking similarities to RPE alterations with age and dysfunctions observed in AMD, which are associated with macrophage infiltration [2] and likely chronic TNFα exposure [14–19]. Our results therefore suggest that strategies inhibiting TNFα signaling in RPE cells might help preserve its essential functions and slow degeneration in late AMD.

## Abbreviations

Mϕ: Macrophage
ACTA2: Smooth muscle alpha (a)-2 actin
AMD: Age-related macular degeneration
CFH: Complement factor H
CFSE: Carboxyfluorescein succinimidyl ester
CLD19: Claudin 19
CNV: Choroidal neovascularization
DMEM: Dulbecco Modified Eagle Medium
EDTA: Ethylenediaminetetraacetic acid
EDU: 5-ethynyl-2′-deoxyuridine
ERG: Electroretinogram
FCS: Fetal calf serum
GA: Geographic atrophy
Mo: Monocyte
OCLD: Occludin
OTX2: Orthodenticle homeobox 2
PBS: Phosphate buffered saline
PFA: Paraformaldehyde
POS: Photoreceptor outer segment
PS: Penicillin streptomycin
qPCR: Quantitative polymerase chain reaction
ROS: Rod outer segments
RPE: Retinal pigment epithelium
RPE65: Retinal pigment epithelium specific *65* kDa protein
RT: Reverse transcription
TER: Trans-epithelial resistance
TGFβ: Transforming growth factor beta
TJ: Tight junction
TNFα: Tumor necrosis factor alpha
TTR: Transthyretin
ZO1: Zonula occludens 1

## References

[1] Strauss O (2005). The retinal pigment epithelium. Physiol Rev.

[2] Guillonneau X, Eandi CM, Paques M, Sahel J-A, Sapieha P, Sennlaub F (2017). On phagocytes and macular degeneration. Prog Retin Eye Res.

[3] Chen M, Rajapakse D, Fraczek M, Luo C, Forrester JV, Xu H (2016). Retinal pigment epithelial cell multinucleation in the aging eye—a mechanism to repair damage and maintain homoeostasis. Aging Cell.

[4] Starnes AC, Huisingh C, McGwin G, Sloan KR, Ablonczy Z, Smith RT (2016). Multi-nucleate retinal pigment epithelium cells of the human macula exhibit a characteristic and highly specific distribution. Vis Neurosci.

[5] Al-Hussaini H, Schneiders M, Lundh P, Jeffery G (2009). Drusen are associated with local and distant disruptions to human retinal pigment epithelium cells. Exp Eye Res.

[6] Flamendorf J, Agrón E, Wong WT, Thompson D, Wiley HE, Doss EL (2015). Impairments in dark adaptation are associated with age-related macular degeneration severity and reticular pseudodrusen. Ophthalmology.

[7] Owsley C, Jackson GR, Cideciyan AV, Huang Y, Fine SL, Ho AC (2000). Psychophysical evidence for rod vulnerability in age-related macular degeneration. Invest Ophthalmol Vis Sci.

[8] Combadière C, Feumi C, Raoul W, Keller N, Rodéro M, Pézard A (2007). CX3CR1-dependent subretinal microglia cell accumulation is associated with cardinal features of age-related macular degeneration. J Clin Invest.

[9] Lad EM, Cousins SW, Van Arnam JS, Proia AD (2015). Abundance of infiltrating CD163+ cells in the retina of postmortem eyes with dry and neovascular age-related macular degeneration. Graefes Arch Clin Exp Ophthalmol.

[10] Levy O, Calippe B, Lavalette S, Hu SJ, Raoul W, Dominguez E (2015). Apolipoprotein E promotes subretinal mononuclear phagocyte survival and chronic inflammation in age-related macular degeneration. EMBO Mol Med..

[11] Sennlaub F, Auvynet C, Calippe B, Lavalette S, Poupel L, Hu SJ (2013). CCR2(+) monocytes infiltrate atrophic lesions in age-related macular disease and mediate photoreceptor degeneration in experimental subretinal inflammation in Cx3cr1 deficient mice. EMBO Mol Med..

[12] Gupta N, Brown KE, Milam AH (2003). Activated microglia in human retinitis pigmentosa, late-onset retinal degeneration, and age-related macular degeneration. Exp Eye Res.

[13] Sedger LM, McDermott MF (2014). TNF and TNF-receptors: from mediators of cell death and inflammation to therapeutic giants—past, present and future. Cytokine Growth Factor Rev.

[14] Bruunsgaard H, Skinhøj P, Pedersen AN, Schroll M, Pedersen BK (2000). Ageing, tumour necrosis factor-alpha (TNF-alpha) and atherosclerosis. Clin Exp Immunol.

[15] Paolisso G, Rizzo MR, Mazziotti G, Tagliamonte MR, Gambardella A, Rotondi M (1998). Advancing age and insulin resistance: role of plasma tumor necrosis factor-alpha. Am J Phys.

[16] Cao S, Ko A, Partanen M, Pakzad-Vaezi K, Merkur AB, Albiani DA (2013). Relationship between systemic cytokines and complement factor H Y402H polymorphism in patients with dry age-related macular degeneration. Am J Ophthalmol.

[17] Agrawal A, Agrawal S, Cao J-N, Su H, Osann K, Gupta S (2007). Altered innate immune functioning of dendritic cells in elderly humans: a role of phosphoinositide 3-kinase-signaling pathway. J Immunol.

[18] Alvarez-Rodríguez L, López-Hoyos M, Muñoz-Cacho P, Martínez-Taboada VM (2012). Aging is associated with circulating cytokine dysregulation. Cell Immunol.

[19] Cousins SW, Espinosa-Heidmann DG, Csaky KG (2004). Monocyte activation in patients with age-related macular degeneration: a biomarker of risk for choroidal neovascularization?. Arch Ophthalmol.

[20] Arnault E, Barrau C, Nanteau C, Gondouin P, Bigot K, Viénot F (2013). Phototoxic action spectrum on a retinal pigment epithelium model of age-related macular degeneration exposed to sunlight normalized conditions. PLoS One.

[21] Law A-L, Parinot C, Chatagnon J, Gravez B, Sahel J-A, Bhattacharya SS (2015). Cleavage of Mer tyrosine kinase (MerTK) from the cell surface contributes to the regulation of retinal phagocytosis. J Biol Chem.

[22] Mathis T, Housset M, Eandi C, Beguier F, Touhami S, Reichman S (2017). Activated monocytes resist elimination by retinal pigment epithelium and downregulate their OTX2 expression via TNF-α. Aging Cell.

[23] Shirasawa M, Sonoda S, Terasaki H, Arimura N, Otsuka H, Yamashita T (2013). TNF-α disrupts morphologic and functional barrier properties of polarized retinal pigment epithelium. Exp Eye Res.

[24] Sullivan DE, Ferris M, Nguyen H, Abboud E, Brody AR (2009). TNF-alpha induces TGF-beta1 expression in lung fibroblasts at the transcriptional level via AP-1 activation. J Cell Mol Med.

[25] Saika S, Kono-Saika S, Tanaka T, Yamanaka O, Ohnishi Y, Sato M (2004). Smad3 is required for dedifferentiation of retinal pigment epithelium following retinal detachment in mice. Lab Investig J Tech Methods Pathol.

[26] Eandi CM, Charles Messance H, Augustin S, Dominguez E, Lavalette S, Forster V, et al. Subretinal mononuclear phagocytes induce cone segment loss via IL-1β. elife. 2016;5. 10.7554/eLife.16490. 10.7554/eLife.16490 PMC4969036 27438413

[27] Moritz T, Niederle N, Baumann J, May D, Kurschel E, Osieka R (1989). Phase I study of recombinant human tumor necrosis factor alpha in advanced malignant disease. Cancer Immunol Immunother CII.

[28] Kutty RK, Samuel W, Boyce K, Cherukuri A, Duncan T, Jaworski C (2016). Proinflammatory cytokines decrease the expression of genes critical for RPE function. Mol Vis.

[29] Zech JC, Pouvreau I, Cotinet A, Goureau O, Le Varlet B, de Kozak Y (1998). Effect of cytokines and nitric oxide on tight junctions in cultured rat retinal pigment epithelium. Invest Ophthalmol Vis Sci.

